# Stress and Alarmins. Report from the 9th iD&EAs meeting

**DOI:** 10.1038/s41419-019-2165-1

**Published:** 2019-12-09

**Authors:** Rosanna Mezzapelle, Emilie Venereau, Marco E. Bianchi

**Affiliations:** 1 Faculty of Medicine, San Raffaele University, Milan, Italy; 2 Division of Genetics and Cell Biology, IRCCS San Raffaele Scientific Institute, Milan, Italy

**Keywords:** Cell death and immune response, Predictive markers

Masahiro Nishibori, Tadatsugu Taniguchi and Ikuro Maruyama organized the International DAMP & Alarmins meeting over 3 sun-drenched days (November 6–9, 2019) in Okayama (Japan). This was the 9th iteration (and the first held in Asia) of the conference series that started in Saltsjöbaden (Sweden) in 2003 and deals with Damage Associated Molecular Patterns (DAMPs) —molecules that are released from dead cells and trigger inflammatory and immune responses— and Alarmins —molecules that are secreted by living cells to the same end: alerting the immune system to danger and stress. Indeed, this conference series defined the field of sterile inflammation, i.e. inflammation that does not arise from infection but is triggered by mechanically damaged or malfunctioning cells.

## DAMPs as misplaced molecules

Several molecules are both DAMPs and alarmins, and these terms are often used interchangeably. DAMPs were initially hypothesized by Polly Matzinger in the late 1990s, as counterparts of Pathogen Associated Molecular Patterns (PAMPs). DAMPs are molecules normally contained in intact cells that are spilled outside the cell following its untimely demise, providing evidence of immediate and actual danger. HMGB1 (High Mobility Group Box 1 protein) was the first such molecule experimentally identified, in 2002. Indeed, HMGB1 is also an alarmin because it can be secreted by stressed cells, via a private secretion pathway that does not involve the endoplasmic reticulum, the Golgi apparatus and secretory vesicles. Other molecules that qualify as DAMPs are ATP, histones and RNA/DNA, which are contained in all cells, and transcription factors and small proteins of the S100 family that are more cell type-specific. Remarkably, all save RNA/DNA are also secreted via private pathways.

DNA that is “misplaced” in the cytoplasm instead of being held inside the nucleus acts as a DAMP, triggering the cGAS-STING pathway and a host of antiviral and immune responses. The same happens with RNAs with structural differences from mRNA and stable RNAs in the cell. Gunther Hartmann (Bonn) provided an overview of the burgeoning field of Nucleic Acid Immunity and presented new evidence on the involvement of RNaseT2 and RNase2 in the processing of RNA to degradation products detected by TLR8, a specific intracellular Toll-like receptor which is only present in primates. Tadatsugu Taniguchi (Tokyo) identified the small nuclear RNA U11 —a component of a minor class of spliceosomal RiboNuclear Particles (snRNPs)— as a ligand of TLR7, a paralog of TLR8. While mature U11 is methylated on many 2’OH, and is not recognized by TLR7, unmethylated U11 is. Modification of fragments of unmethylated U11 with phosphorothioates produces very strong TLR7 agonists, whereas methylated derivatives of U11 provide potent TLR7 antagonists for potential therapeutic approaches.

## New DAMPs and receptors

In addition to being numerous themselves, DAMPs have numerous post-translational modifications and a multitude of interacting proteins and receptors. Sho Yamasaki (Osaka) showed that Mincle2, a receptor for mycobacterial and fungal PAMP glycolipids, is also activated by the DAMP glycolipid β-glucosylceramide (β-GlcCer), which is released by dead cells. Remarkably, mutations that increase the basal level of β-GlcCer cause a form of Gaucher’s disease, a devastating neuroinflammatory disease.

Johannes Roth (Münster) reported an example of DAMPs promoting inflammation in one form and restricting it in an alternative form. Dimers of S100A8/A9 activate TLR4 and RAGE, but calcium ions promote their self-association into tetramers, which no longer can bind TLR4 or RAGE but dampen inflammation by binding CD69 in macrophages and restricting cytoskeleton dynamics. S100 proteins can also bind to RAGE paralogs MCAM, ALCAM, EMMPRIN, NPT*α* and NPT*β*, reported Masakiyo Sakaguchi (Okayama). All these receptors tend to be elevated in cancer and favour metastasis.

Hang Hubert Yin (Beijing) discovered a new receptor for HMGB1: TLR5, better known as the flagellin receptor. TLR5 recognizes the acidic tail of HMGB1 and induces allodynia. Jaroslaw Zmijewski (Birmingham, USA) characterized a new post-translational modification of HMGB1, and a cognate interacting protein: HMGB1 glutathionylated on C106 binds to gp91, a subunit of NOX2, and reduces ROS production by neutrophils; fully reduced HMGB1 does not bind or modulate NOX2. Angelo Manfredi (Milan) showed that HMGB1 can be associated to microvesicles emitted by platelets, which bind and activate neutrophils. HMGB1-bearing vesicles are extremely abundant in the blood of scleroderma patients, and when injected in mice recapitulate some aspects of scleroderma.

## DAMP trafficking

Understanding how DAMPs are actively secreted may unveil new drug targets. Seung Bum Park (Seoul) argued that inflachromene, the anti-neuroinflammation molecule that he synthesized, blocks HMGB1 and -2 secretion by making them refractory to acetylation by nuclear acetyltransferases. Hiroshi Ueda (Kyoto) reported that amlexanox inhibits the secretion of the DAMP prothymosin-*α*, which is driven to the extracellular side of the plasma membrane by ANXA2.

HMGB1 getting out of living cells is only the flip coin of the story, though. Ben Lu (Changsha) first showed that HMGB1 can re-enter monocytes bringing LPS along. The HMGB1–LPS complex arrives to lysosomes, and blows them open when they become acidic. Thus liberated in the cytoplasm, LPS activates Caspase 11, triggering inflammasome activation, gasdermin D cleavage and pyroptosis, and promotes sepsis. He illustrated how the Casp-11 pathway is involved in Disseminated Intravascular Coagulopathy (DIC). In DIC, coagulation is largely driven by tissue factor (TF) rather than thrombin or platelet coagulation. Gasdermin D forms pores in the plasma membrane that allow calcium entry, activating the scramblase TMEM16F, which flips phosphatidylserine to the outer surface of the plasma membrane, where it engages and dramatically activates TF. Anna Rubartelli (Genoa) introduced the notion of a graded response of monocytes to inflammatory stimuli: at lower stimulation, monocytes secrete IL-1*β* via secretory lysosomes but do not die; at higher stimulation, they activate the inflammasome and undergo pyroptosis.

Consistent with all these results, Tim Billiar (Pittsburgh) showed single cell transcriptomics of bone marrow and peripheral blood from mice with trauma, which indicate that monocytes are the cells that respond the most, and with specific signatures.

## A special session on scientific fraud perpetrated by Daniel J. Antoine

Ulf Andersson (Stockholm) reported on a shocking case of scientific fraud that has rocked the DAMP field. HMGB1 acetylation was identified in 2003 by M.E. Bianchi. Daniel Antoine, then at the University of Liverpool, reported in 2009 that HMGB1 acetylation could be accurately quantified by mass spectrometry. He soon became the expert with whom to collaborate, and contributed to dozens of papers. However, in 2017 he stopped responding to calls relating to his results. The University of Liverpool started an investigation into his activities, and he resigned from his position at Edinburgh, where he had just moved, and disappeared. Although the investigation is not complete yet, it is now obvious that many of his results were fabricated. All available evidence indicates that Dr. Antoine acted alone; his co-authors were not in any way aware of his fabrications.

Since his laboratory notebooks are not retrievable, the participants to the conference agreed that D.J. Antoine’s entire scientific output is now suspect and should not be relied upon. They also agreed that his quantitative data confirm experimental results first obtained by others, using different approaches. HMGB1 post-translational modifications do exist and they do matter; his mass spectrometry methods probably don’t. Despite his egregious blow to scientific integrity and to the professional lives of his junior colleagues, it is safe to conclude that in most cases the removal of his data (whether fabricated or not) does not alter the validity of the conclusions reached in the publications involved.

## DAMPs meet the brain

Ischemia induces DAMP secretion, and consequently DAMPs play a large role in stroke, as initially showed by Masahiro Nishibori. Akihiko Yoshimura (Tokyo) showed that peroxiredoxin-5 and -6, abundant and ubiquitous redox proteins that can be considered DAMPs, are released during stroke and activate microglia via TLR2 and -4. DAMPs, including HMGB1, are cleared by macrophages via Marco and Msr-1 scavenger receptors. Stefan Roth (Munich) showed that HMGB1 release after stroke markedly increases the occurrence of atherosclerosis, which can be counteracted with soluble RAGE. His unpublished results indicate that DAMPs also cause a dramatic disappearance of T cells from lymphoid organs, and thus immune depression. This is due to a crosstalk between macrophages (expressing FasL and IL-1*β*) and lymphocytes (expressing Fas).

HMGB1 also appears to be involved in various neurodegenerative diseases, such as Parkinson and Alzheimer Disease (AD). Hitoshi Okazawa (Tokyo) showed that administration of an anti-HMGB1 monoclonal antibody after the onset of AD slows down progression.

Atsufumi Kawabata (Osaka), Yoki Nakamura (Kyoto) and Huan Yang (Manhasset) all reported that HMGB1 is involved in neuropathic pain (NP), which affects 7–8% of adults. Kawabata focused on chemotherapy-induced peripheral neuropathy (CIPN), a common side effect of cancer chemotherapy. CIPN is accompanied by ROS production, activation of nuclear acetylases, NF-kB and p38 MAPK, and HMGB1 release. Human soluble thrombomodulin, a drug approved in Japan for patients with DIC, promotes HMGB1 proteolytic inactivation by thrombin and ameliorates CIPN. Conversely, anticoagulants that reduce thrombin activation all increase HMGB1 and CIPN. Nakamura showed that HMGB1 increases in constricted sciatic nerve, a model of NP. Correspondingly, peri-sciatic nerve injection of HMGB1 activates microglia and induces NP, which can be reduced by minocycline and fluorocitrate, inhibitors of microglia and astrocyte activation, respectively. Depression is a common comorbidity of NP; anti-HMBG1 mAb and glycyrrhizin (a HMGB1 inhibitor) can ameliorate depression. Yang used sophisticated inducible mouse models to show that the source of HMGB1 in NP are neurons.

Tomoyuki Furuyashiki (Kobe) initially discovered that TLR2 and -4 are activated in mouse microglia during the repeated social defeat-induced depression. A mouse is repeatedly exposed to a much larger and aggressive mouse, and this frustrating confrontation leads it into a state of social avoidance (reduced interaction with an unacquainted mouse) and increased anxiety (increased tendency to avoid open arms in an elevated plus maze). Such depressive behaviour correlates to neurite atrophy in the prefrontal cortex, where social behavioural inputs are processed. Mice that have been injected with a viral vector leading to TLR2/TLR4 double knockdown in the microglia of the prefrontal cortex are resistant to defeat-induced depression. TLR2 and TLR4 are both receptors for HMGB1, and Furuyashiki’s unpublished data indicate that HMGB1 is translocated from the nucleus to the cytoplasm of prefrontal neurons and promotes depressive behaviour.

## Conclusions

We are now attaining a clearer understanding of sepsis, trauma and sterile inflammation, and new inhibitors are emerging that could become life-saving drugs. Some, like soluble human thrombomodulin, are already approved drugs. The homeostatic functions of DAMPs, however, are less well understood.

The most stunning insight provided by this meeting is that stress, as defined in the psychological and psychiatric domains, depends on molecules that are key in inflammation and immunity: DAMPs and their receptors.

The 10th iD&As meeting will be held in Saltsjöbaden (Sweden) on June 9–11, 2021. We predict that, by then, we will have seen an amazing blooming of the DAMP field. Visionaries please apply now.

